# Molecular Taxonomy of Geckos Reveals a Second *Tarentola* Species (Reptilia: Squamata) on the Maltese Islands

**DOI:** 10.3390/genes17030271

**Published:** 2026-02-26

**Authors:** Noel Vella, Marina Zorrilla García, Adriana Vella

**Affiliations:** Conservation Biology Research Group, Department of Biology, Faculty of Science, University of Malta, MSD2080 Msida, Malta; noel.vella@um.edu.mt (N.V.);

**Keywords:** 12S, 16S, COI, DNA barcoding, *Hemidactylus turcicus*, *Tarentola mauritanica*, *Tarentola fascicularis*/*deserti*

## Abstract

**Background/Objectives**: The Maltese islands, situated in the Sicilian Channel, are known to host two gecko species, *Hemidactylus turcicus* and *Tarentola mauritanica*. However, gecko taxonomy is complicated by cryptic lineages within species complexes, requiring molecular approaches for accurate identification. **Methods**: In this study, we investigated species diversity using opportunistic sampling of 30 dead gecko specimens, including road-killed individuals, from across the Maltese islands. Due to the degraded condition of most samples, morphological identification was limited; therefore, mitochondrial markers (12S, 16S and COI) were employed to assign species identity. **Results**: Our analyses revealed the first records of the *Tarentola fascicularis*/*deserti* complex in Malta. This finding extends the known distribution of this complex and complements records from neighbouring islands in the Sicilian Channel, where *T. mauritanica* and *T. fascicularis*/*deserti* lineages occur in sympatry. **Conclusions**: Given the greater ecological affinity of the *T. fascicularis*/*deserti* complex for arid environments, these findings emphasise the importance of continued monitoring to clarify the dynamics of sympatry, potential ecological displacement, and the long-term effects of climate change and anthropogenic activity on the central Mediterranean herpetofauna.

## 1. Introduction

The Maltese islands are known to host two species of geckos, *Hemidactylus turcicus* (Linnaeus, 1758) (Squamata: Gekkonidae) and *Tarentola mauritanica* (Linnaeus, 1758) (Squamata: Phyllodactylidae) [1,2,3,4]. Gecko genera are typically characterised by conserved morphology with contrasting molecular data that reveal substantial genetic diversity, often exhibited in species complexes [5,6,7,8,9,10,11,12,13,14]. Currently, there are around 200 species within the genus *Hemidactylus* Oken, 1817 [3,15], with *H. turcicus* being found in all Mediterranean countries [3,16]. The genus *Tarentola* Gray, 1825 currently comprises 33 recognised species [3]. Phylogenetic analyses of the *T. mauritanica* complex show that this taxon is paraphyletic, with respect to *T. angustimentalis*, and forms six clades (Clades I–VI sensu Rato et al. [12]), each supporting a putative species [10]. Phylogenetic analyses of *Tarentola fascicularis* and *Tarentola deserti* indicate that these two species form the *T. fascicularis*/*deserti* complex composed of nine clades (Clades VII–XV sensu Rato et al. [12]). The *T. mauritanica* complex is widely distributed across Mediterranean countries [3,16], whereas the *T. fascicularis*/*deserti* complex occurs mainly in North Africa (Morocco, Algeria, Tunisia, Libya and Egypt) and on islands of the Sicilian Channel [8,12,17,18] and seems to be more prevalent in drier habitats than *T. mauritanica* [19]. Until now, this complex has not been detected on the islands of Linosa and Sicily and across mainland southern Europe [12,20,21]. It was thought to be absent in Malta, as the only *Tarentola* species historically recorded here was *T. mauritanica*, based on morphological characteristics [1,2,4,22,23] and the genetic analysis of two specimens [21].

The current diversity and distribution of terrestrial reptiles in the Mediterranean have been shaped by major historical geographical events, including the Messinian Salinity Crisis [24] and the more recent glacial cycles that caused shifts in climate and sea levels [25,26,27]. These changes altered habitats and connectivity between land masses, leading to the formation of temporary land bridges and refugia that influenced the migration and evolutionary trajectories of terrestrial fauna in the region [12,20,28]. Additionally, dispersal by rafting, as observed in other gecko taxa [29], may have further contributed to their distribution. In more recent times, human-mediated dispersal has further contributed to shaping the composition of reptile diversity and lineages in the region [28,30,31,32]. Within this scenario, the Maltese archipelago, located approximately 85 km south of Sicily, 150 km northeast of Lampedusa, and 300 km north of Tunisia provides an important setting for ecological and biogeographic studies. Its isolation has led to unique evolutionary paths, including endemic diversity at both species and genetic levels [33,34]. During glacial cycles, sea-level fluctuations created temporary land bridges between Malta and nearby islands, namely the Hyblaean region in Sicily, facilitating faunal exchange [27,35], while human-mediated transportation may have further contributed to species dispersal [22]. The terrestrial reptile fauna of the Maltese archipelago comprises ten species, including the introduced and now naturalised *Chamaeleo chamaeleon* and the more recently introduced *Indotyphlops braminus* [1,2,4,36].

Within this biogeographic context, geckos were selected to investigate patterns of diversity and lineage composition in the Maltese islands. As *H. turcicus* and *T. mauritanica* are strictly protected under Maltese law [37], only dead specimens, mainly road-killed individuals, were examined. Most specimens were in poor morphological condition, often highly degraded with missing body parts, thus lacking diagnostic features, which limited morphological identification. Additionally, given the prevalence of cryptic lineages in gecko taxa, molecular taxonomy was therefore required for reliable species delimitation. Analyses focused on the mitochondrial 12S rRNA gene (12S) and 16S rRNA gene (16S), which are widely used in Mediterranean gecko studies [7,10,11,12,18,20], as well as on cytochrome c oxidase subunit I (COI), a marker that is rarely applied to *Tarentola* but used extensively as a DNA barcode for species identification [38,39,40].

## 2. Materials and Methods

**Figure 1:**
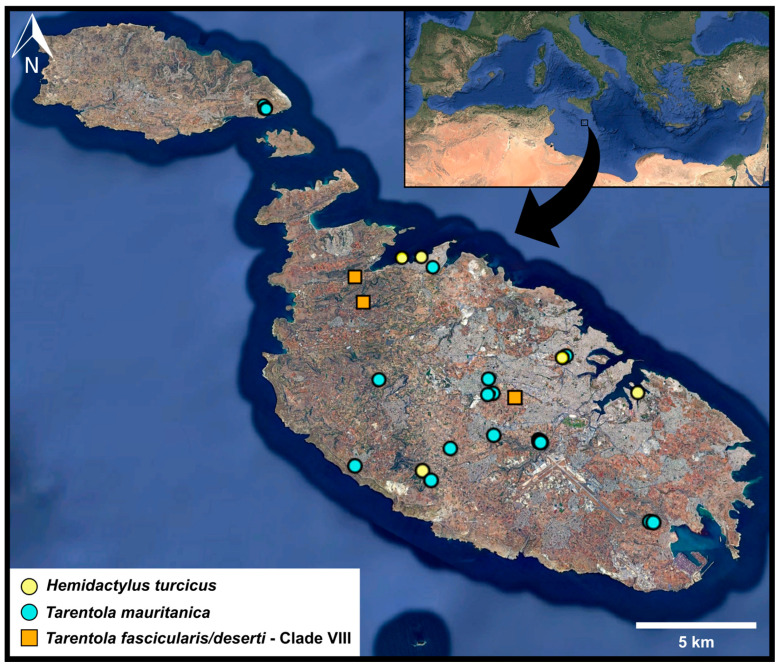
Map of collection sites [44]. Some points represent locations where multiple specimens were sampled (Table 1). Squares represent records of the newly detected *Tarentola fascicularis*/*deserti* species in Malta.

This study forms part of ongoing biodiversity monitoring conducted by the Conservation Biology Research Group, University of Malta. Targeted sampling sessions were carried out on foot from April to September in 2020 and from May to August in 2025. Thirty dead gecko specimens were found at various locations around the Maltese islands (Figure 1; Table 1), and each was sampled following local Environmental and Resource Authority permits. The collected tissue samples were kept frozen at −20 °C until analysed.

Genomic DNA was extracted from the collected tissue samples using the GF-1 Tissue DNA Extraction Kit (Vivantis Technologies, Shah Alam, Malaysia), and the concentration of the purified DNA was estimated using a Qubit fluorometer (ThermoFisher Scientific, Waltham, MA, USA). The mtDNA genes amplified were: 12S using primers 12SAL and 12SBH, following Kocher et al. [41]; 16S using primers 16Sar-L and 16Sbr-H, following Palumbi [42]; and COI, using either primers REPCOI-F and REPCOI-R, following Nagy et al. [39], or primers jgLCO and jgHCO, following Geller et al. [43].

Each PCR product was purified and sequenced using an ABI3730XL sequencer (Applied Biosystems Waltham, MA, USA), using the respective forward and reverse primers. Sequences were checked for consistency using Geneious v10 [45]. As the specimens were not morphologically identified, the resulting sequences were compared with publicly available data through BLASTn (https://www.ncbi.nlm.nih.gov) [46] and BOLD (http://www.boldsystems.org) [47,48] for molecular and barcode index number (BIN) identification. Diversity indices for the sampled specimens were estimated using Arlequin v3 [49] (Table A1). Sequences were aligned using Geneious v10 [45]. Phylogenetic analysis of the gecko specimens sampled in this study was carried out using homologous data from *C. chamaeleon* (EF222201 [50]) as an outgroup. The phylogenetic tree was constructed using Bayesian Inference via MrBayes v3.2.6 [51] within Geneious v10, with 1 × 10^7^ generations, a sampling frequency of every 2000 generations and a burn-in of 25%, using the best-fit substitution model identified through jModelTest2 [52]. Intraspecific relationships among haplotypes were visualised by constructing minimum spanning networks using TCS v1.21 [53]. Phylogenetic analyses of the currently studied *T. mauritanica* and *T. fascicularis*/*deserti* specimens in relation to other members of the same species complexes were performed using genetic data as indicated in Table A2 and Table A3, with *T. neglecta* (JQ300874), the closest related species to the *T. fascicularis*/*deserti* complex [12], being used as an outgroup. These analyses were conducted using the same Bayesian Inference parameters described above.

## 3. Results

**Figure 2:**
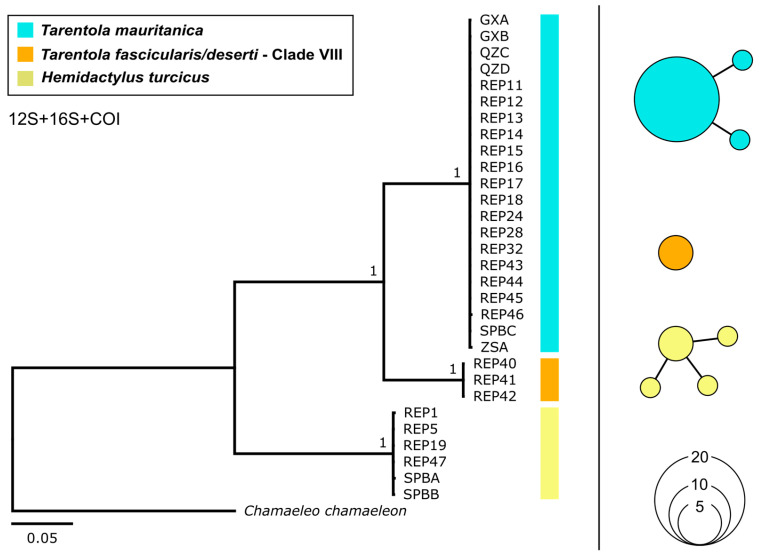
Phylogenetic tree inferred using Bayesian Inference for the 30 gecko specimens collected from Malta, based on the three concatenated loci, with *Chamaeleo chameleon* (EF222201 [50]) as an outgroup (**left**). Haplotype networks for each respective species, based on concatenated data (**right**).

Sequences generated during this study were deposited in GenBank (Table 1). Phylogenetic analyses resolved the 30 analysed specimens into three well-supported groups, each showing sufficient genetic divergence across the three loci, justifying the recognition of three distinct species of geckos in Malta (Figure 2). Sequence matches with GenBank and BOLD identified *H. turcicus* (*n* = 6), *T. mauritanica* (Clade III sensu Rato et al. [12]) (*n* = 21), and the first Maltese records of *T. fascicularis*/*deserti* (Clade VIII sensu Rato et al. [12]) (*n* = 3) (Table 1). The COI data showed the greatest divergence among the examined taxa (Figure 2). The pairwise differences between *T. mauritanica* and *T. fascicularis*/*deserti* in this study were 9.0% for 12S, 11.3% for 16S and 17.4–17.6% for COI.

### 3.1. Hemidactylus turcicus (Linnaeus, 1758)

Six specimens were collected near human-built environment (Table 1). These specimens exhibited one haplotype for 12S (393 bp), three haplotypes for 16S (647 bp) and two haplotypes for COI (561 bp), with the concatenated data producing four distinct haplotypes (Figure 2; Table A1). The 12S sequences showed a 100% identity match with conspecifics, with a 100% similarity to MZ388481 (specimen DB31624), which forms part of the Europe and North African clade identified in Rato et al. [54].

Specimens REP5, REP19, SPBA and SPBB shared the same 16S haplotype, which differed by 1 bp from the haplotypes of REP1 and REP47. These haplotypes showed 99.8% and 99.7% identity with MT378392 (*H. turcicus* from USA); however, it is noteworthy that 16S data for this species is currently less extensive than 12S data. These sequences exhibited high identity (99.8% to 100%) with haplotypes previously recorded from Sicily (Italy) and northern Tunisia. In contrast, sequence identity with the southern mainland Italy and Linosa (Italy) clade identified by Stöck et al. [21] was notably lower, ranging between 98.9% and 99.3%. Five specimens shared an identical COI haplotype, while specimen SPBA exhibited a 1 bp divergence. All COI haplotypes showed 99.6% to 99.8% similarity with conspecifics.

Molecular identification through BOLD assigned all specimens to BIN BOLD:ABY0780, a cluster that includes representatives of *H. turcicus* from its invasive range in the United States. This BIN exhibited an average internal *p*-distance of 0.12% (maximum 0.33%) and a 2.40% *p*-distance to its nearest neighbour (BOLD:AAX1357), which is also composed of *H. turcicus* lineages.

### 3.2. Tarentola mauritanica (Linnaeus, 1758)

Twenty-one specimens were analysed, primarily collected from agricultural land and urban fringes (Table 1). These specimens exhibited a single haplotype for 12S (383 bp), a single haplotype for 16S (399 bp), and three haplotypes for COI (638 bp) (Table A1). Nineteen specimens shared the same COI sequence, while REP46 and ZSA differed by 1 bp.

**Figure 3:**
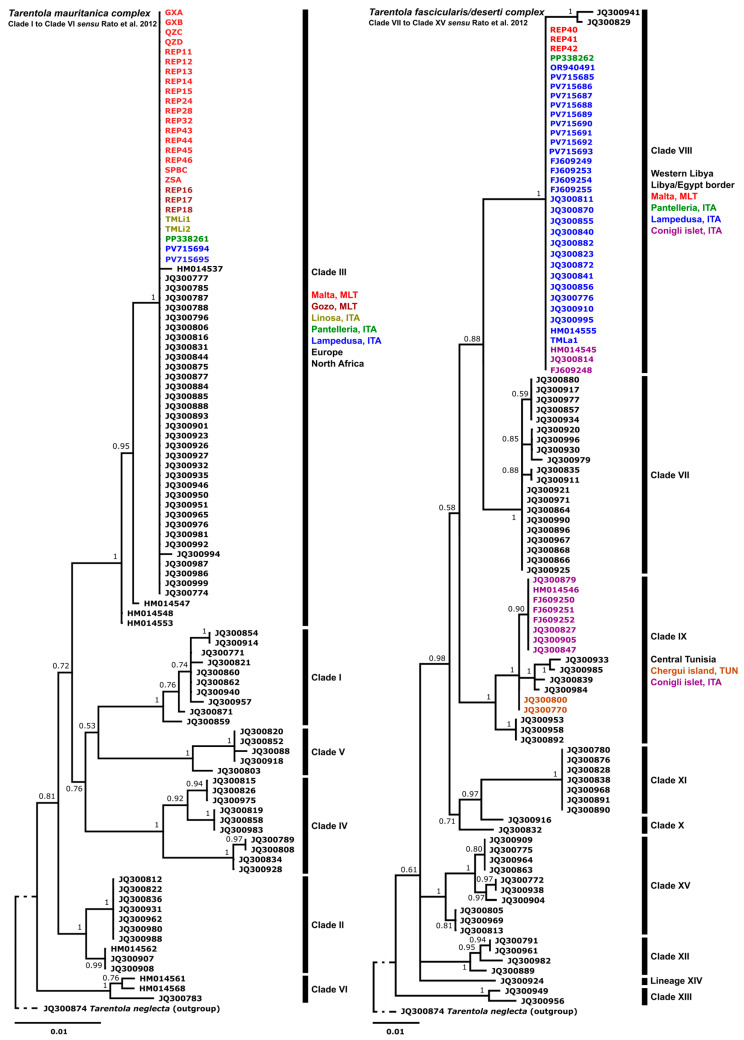
Phylogenetic trees using Bayesian Inference analyses 16S data, focusing on *Tarentola mauritanica* complex (**left**) and *Tarentola fascicularis*/*deserti* complex (**right**). Analyses include data from other published works [8,12,17,18,20,21,56], and clade assignment followed Rato et al. [12]. Locations have been added to clades represented on islands in the Sicilian Channel (Table A2 and Table A3).

The 12S, the 16S and the most common COI sequence showed 100% identity with JQ425060 (*T. mauritanica* DB11105 representing the European lineage from Italy [55]). Phylogenetic comparisons of the 12S and 16S data placed the Maltese specimens within Clade III of the *T. mauritanica* complex, as defined by Rato et al. [12] (Figure 3). The 16S sequence of the Maltese specimens had a 100% match to those analysed from Linosa, Lampedusa (Italy), Pantelleria (Italy) and Sicily, mainland Europe and north Africa [12,17,18,21,56]. All our *T. mauritanica* specimens were assigned to BOLD:AAK0390, which is composed of specimens collected mainly from Italy and Spain (within BIN: mean *p*-distance 0.31%; maximum *p*-distance 0.97%). This BIN is separated by a 5.26% *p*-distance from its nearest neighbour (BOLD:ACH7590), supporting its clear divergence within the species complex.

### 3.3. Tarentola fascicularis/deserti (Clade VIII, sensu Rato et al. [12])

Three specimens were collected near agricultural land (Table 1). Two specimens were found in September 2020 and one specimen in May 2025 and represent the first records for this complex in the Maltese islands. These specimens exhibited a single haplotype for all three examined loci (12S—399 bp; 16S—526 bp; COI—658 bp). The 12S and 16S sequences exhibited 100% identity with reference sequences HM014488 and HM014545, respectively, which were both derived from specimen DB303, collected at Conigli Islet, Italy [56]. Our 16S sequence was also an identical match to the recently published sequences PP338262 from Pantelleria [17] and OR940491 from Lampedusa [20]. The COI data yielded no high-identity matches on BOLD and GenBank due to the lack of publicly available COI barcodes for this clade (Table A1).

**Figure 4:**
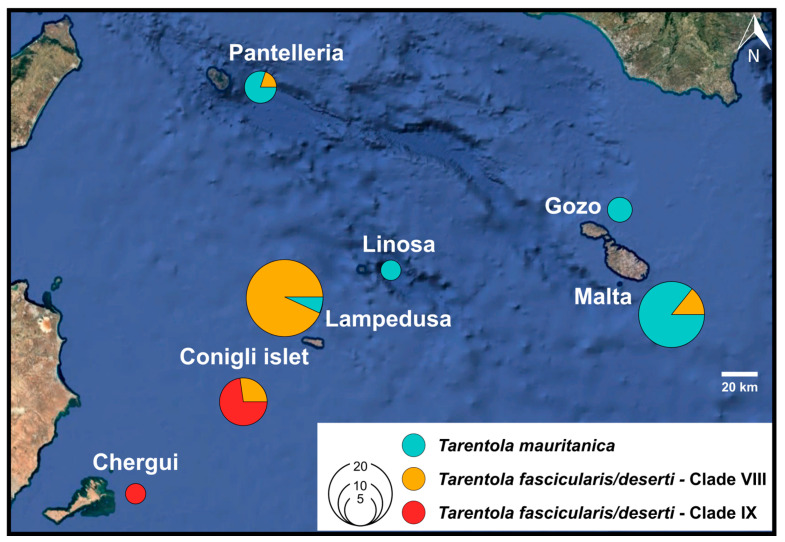
Map of the Sicilian Channel [44] showing the islands and the distribution of the different *Tarentola* clades identified in the region, based on 12S and/or 16S sequences. Data for the Maltese specimens were obtained from the present study, while information for the other islands comes from the studies listed in Table A2 and Table A3.

Comparative analyses of 16S sequences with published data for this complex (Figure 3; Table A2) confirm that the Maltese specimens cluster within Clade VIII of the *T. fascicularis*/*deserti* complex (sensu Rato et al. [12]). This clade encompasses populations from the Pelagian Islands (Lampedusa and Conigli islet) and western Libya, extending toward the Egyptian border. Clade VIII is a sister to Clade VII (*T. deserti*) (Figure 3). Given that the Maltese haplotypes are distinct from Clade VII and Clade XI (the latter containing the *T. fascicularis* type locality), and given the cryptic diversity within the genus, we assign these specimens to *T. fascicularis*/*deserti* Clade VIII. This classification is consistent with Mori et al. [18], who identified the same 16S haplotype in Lampedusa populations (Figure 3 and Figure 4). Notably, while the Maltese and Pelagian island populations share an identical 16S sequence, they exhibit a 1.1% to 1.6% sequence divergence from mainland Libyan individuals within the same clade (Figure 3).

## 4. Discussion

This study uses genetic evidence to confirm the presence of three gecko species on the Maltese islands and provides new insights into the genetic diversity of *H. turcicus* and *T. mauritanica.* While the latter two species are well-documented in the archipelago [1,2,4], this study identifies previously unrecorded haplotypes and links them to specific lineages. Additionally, we report the first records of *T. fascicularis*/*deserti* (Clade VIII sensu Rato et al. [12]) in Malta. Although mitochondrial divergence alone may not always conclusively demonstrate sympatric coexistence of closely related species due to possible hybridisation, nuclear data from other studies support divergence between these lineages using biparentally inherited markers [12,17]. Moreover, mitochondrial divergence between the *Tarentola* species here reached 8.5%, 9.6% and 17.4% for 12S, 16S and COI, respectively, exceeding typical intraspecific variation for these markers [10,39].

The *T. fascicularis*/*deserti* complex has successfully colonised several islands from the Sicilian Channel (Figure 4), where it is represented by two cryptic lineages, Clades VIII and IX sensu Rato et al. [12]. Clade VIII occurs in western Libya, the Libya–Egypt border, Pantelleria and Lampedusa, and is sympatric with Clade IX on the nearby Conigli islet [8,12,17,18,21,56] (Figure 4). Clade IX occurs in central Tunisia, Chergui island (Kerkennah archipelago, Tunisia) and Conigli islet [8,12,56]. The latter two islands host individuals belonging to distinct lineages within Clade IX (Figure 4). Notably, Clade IX has not been detected on mainland Lampedusa, despite its occurrence on the neighbouring Conigli islet [18]. The absence of records of the *T. fascicularis*/*deserti* complex from Linosa and Sicily should be interpreted with caution, as sampling of the genus *Tarentola* is limited. Based on the currently available genetic data, Linosa is represented by only two *Tarentola* specimens [21], while genetic surveys of *Tarentola* in Sicily [20,21] are geographically restricted, lacking extensive coverage of south-eastern regions.

In Malta, *T. fascicularis*/*deserti* is not confined to a particular region, suggesting that it has been present on the archipelago for some time, which is possibly the result of historical introductions. All known European populations of *T. fascicularis*/*deserti* Clade VIII share the same 16S haplotype [8,12,17,18,20,21,56] despite occurring on islands with different geological origins and histories [35,57]. Given the slow evolutionary rate of the 16S gene in reptiles [58,59], divergence since the Late Pleistocene may be insufficient to generate detectable genetic variation. However, the lack of regional haplotype differentiation strongly suggests a common and possibly recent origin. In this context, human-mediated dispersal therefore represents a plausible explanation, considering the substantial faunal input from North Africa [21,22]. Future studies using additional genetic markers may help to clarify these patterns further.

*Tarentola mauritanica* is widely distributed throughout the Mediterranean, with Clade III sensu Rato et al. [12] representing all specimens from the central Mediterranean region, including Sicily and mainland Italy [10,12,20]. The presence of Clade III in Malta reinforces historical biogeographical links with Sicily. Clade III has also been detected on the islands of Linosa [21], Pantelleria [17] and Lampedusa [18], with the latter two islands hosting both *T. mauritanica* Clade III and *T. fascicularis*/*deserti* Clade VIII. On Pantelleria, Antinucci et al. [17] genetically identified four specimens of *T. mauritanica* near the main harbour, and one specimen of *T. fascicularis*/*deserti* within the Pantelleria National Park. On Lampedusa, *T. fascicularis*/*deserti* Clade VIII appears to be the predominant taxon, being widely distributed across the island, with past records consistently reporting individuals of this clade as the sole *Tarentola* species present [12,18,56]. However, recently, two *T. mauritanica* specimens were collected in the port area in Lampedusa, suggesting recent human-mediated introduction [18].

In Malta, *T. mauritanica* is widely distributed, whereas records of the *T. fascicularis*/*deserti* complex are comparatively rare. Ecologically, these two taxa show subtle partitioning [60]. While both are adapted to arid environments, *T. fascicularis* and *T. deserti* generally occupy hyper-arid to arid habitats, whereas *T. mauritanica* is more prevalent in semi-arid and mesic zones [19]. The predominance of *T. mauritanica* may reflect Malta’s relatively higher humidity compared to Lampedusa, although further niche modelling is required. Additionally, as our sampling was derived opportunistically from road-killed individuals, the dataset is potentially biassed toward inhabited areas and transport corridors. This may favour the detection of the synanthropic *T. mauritanica*, potentially overestimating its relative abundance compared to the *T. fascicularis*/*deserti* complex.

The addition of *T. fascicularis*/*deserti* to the Maltese herpetofauna highlights the need for further targeted surveys, including the minor islands and islets (Comino, Filfla and St. Paul’s Islands), where isolated relict populations and possible additional instances of sympatry may occur. Such surveys would clarify this clade’s distribution and help to determine whether it represents a native or long-established cryptic component of the Maltese fauna, a necessary first step prior to any conservation assessment. The absence of this taxon from current Maltese legislation (S.L. 549.44, Environment Protection Act), which protects local native and naturalised reptiles [37], underscores the importance of regularly updating conservation policies to reflect emerging taxonomic and biogeographical knowledge, particularly in insular systems and for taxa in which cryptic diversity is common. Collectively, these findings highlight the need for a comprehensive taxonomic reassessment of the genus *Tarentola*, as several lineages likely warrant formal species-level recognition [10], which would improve the implementation of species-specific conservation measures. Understanding the distribution of these clades is increasingly critical, considering accelerating climate change and intensifying anthropogenic pressures on Mediterranean reptile diversity.

## 5. Conclusions

Our findings expand the known range of *T. fascicularis*/*deserti* Clade VIII sensu Rato et al. [12] to the Maltese archipelago, which is situated approximately 150 km east of Lampedusa. The discovery of a third gecko species in Malta, the second within the genus *Tarentola*, underscores the critical role of genetic monitoring in the management of protected taxa. This study demonstrates how molecular tools can resolve taxonomic ambiguity, even in degraded or morphologically cryptic specimens. By confirming the presence of two distinct *Tarentola* taxa, this research establishes the Maltese islands as a pivotal site for understanding central Mediterranean biogeography and the complex dispersal dynamics of the region’s herpetofauna.

## Figures and Tables

**Table 1:** Data on specimens analysed during this study.

Specimen ID	Date of Sampling	GPS	Location, Island	GenBank Accession Numbers 12S; 16S; COI
***Hemidactylus turcicus* (Linnaeus, 1758)**				
CBRG-REP1	4 August 2020	35°54′7″ N, 14°29′4″ E	Msida, Malta	MZ478108; MZ477454; MZ477390
CBRG-SPBA	9 August 2020	35°56′57″ N, 14°24′2″ E	St. Paul’s Bay, Malta	MZ478111; MZ477457; MZ477393
CBRG-SPBB	9 August 2020	35°56′59″ N, 14°24′40″ E	St. Paul’s Bay, Malta	MZ478112; MZ477458; MZ477394
CBRG-REP19	15 August 2020	36°1′44″ N, 14°19′17″ E	Qala, Gozo	MZ478110; MZ477456; MZ477392
CBRG-REP5	16 August 2020	35°53′10″ N, 14°31′16″ E	Birgu, Malta	MZ478109; MZ477455; MZ477391
CBRG-REP47	3 May 2025	35°51′09″ N, 14°24′41″ E	Siġġiewi, Malta	PX789229; PX788532; PX788521
***Tarentola mauritanica* (Linnaeus, 1758) (Clade III sensu Rato et al. [** 12 **])**				
CBRG-REP11	8 April 2020	35°51′53″ N, 14°28′11″ E	Qormi, Malta	MZ478118; MZ477472; MZ477409
CBRG-REP12	15 April 2020	35°51′52″ N, 14°28′13″ E	Qormi, Malta	MZ478119; MZ477473; MZ477410
CBRG-REP13	18 April 2020	35°51′56″ N, 14°28′10″ E	Qormi, Malta	MZ478120; MZ477474; MZ477411
CBRG-REP14	21 April 2020	35°51′54″ N, 14°28′11″ E	Qormi, Malta	MZ478121; MZ477475; MZ477412
CBRG-REP15	27 April 2020	35°51′53″ N, 14°28′14″ E	Qormi, Malta	MZ478122; MZ477476; MZ477413
CBRG-REP16	22 June 2020	36°1′50″ N, 14°19′15″ E	Qala, Gozo	MZ478123; MZ477477; MZ477414
CBRG-REP17	22 July 2020	36°1′42″ N, 14°19′21″ E	Qala, Gozo	MZ478124; MZ477478; MZ477415
CBRG-SPBC	9 August 2020	35°56′41″ N, 14°25′3″ E	St. Paul’s Bay, Malta	MZ478126; MZ477480; MZ477417
CBRG-REP18	10 August 2020	36°1′42″ N, 14°19′17″ E	Qala, Gozo	MZ478125; MZ477479; MZ477416
CBRG-ZSA	6 September 2020	35°51′43″ N, 14°25′31″ E	Żebbuġ, Malta	MZ478127; MZ477481; MZ477418
CBRG-GXA	27 September 2020	35°49′54″ N, 14°31′16″ E	Birżebbuġa, Malta	MZ478115; MZ477468; MZ477405
CBRG-GXB	27 September 2020	35°49′53″ N, 14°31′22″ E	Birżebbuġa, Malta	PX789233; MZ477469; MZ477406
CBRG-QZC	21 September 2020	35°53′9″ N, 14°26′51″ E	Attard, Malta	MZ478116; MZ477470; MZ477407
CBRG-QZD	21 September 2020	35°53′7″ N, 14°26′41″ E	Attard, Malta	MZ478117; MZ477471; MZ477408
CBRG-REP24	6 June 2025	35°52′03″ N, 14°26′50″ E	Żebbuġ, Malta	PX789234 PX788536; PX788522
CBRG-REP28	6 July 2025	35°53′31″ N, 14°23′22″ E	Rabat, Malta	PX789235; PX788537; PX788523
CBRG-REP32	14 August 2025	35°53′32″ N, 14°26′43″ E	Attard, Malta	PX789236; PX788538; PX788524
CBRG-REP43	6 June 2025	35°54′09″ N, 14°29′08″ E	Msida, Malta	PX789237; PX788539; PX788525
CBRG-REP44	6 June 2025	35°54′09″ N, 14°29′08″ E	Msida, Malta	PX789238; PX788540; PX788526
CBRG-REP45	1 July 2025	35°51′17″ N, 14°22′41″ E	Dingli, Malta	PX789239; PX788541; PX788527
CBRG-REP46	23 July 2025	35°50′55″ N, 14°24′56″ E	Siġġiewi, Malta	PX789240; PX788542; PX788528
***Tarentola fascicularis*/*deserti* (Clade VIII sensu Rato et al. [** 12 **])**				
CBRG-REP40	26 September 2020	35°55′40″ N, 14°22′50″ E	Mġarr, Malta	PX789230; PX788533; PX788529
CBRG-REP41	26 September 2020	35°56′24″ N, 14°22′33″ E	Mġarr, Malta	PX789231; PX788534; PX788530
CBRG-REP42	5 June 2025	35°53′02″ N, 14°27′31″ E	Qormi, Malta	PX789232; PX788535; PX788531

## Data Availability

Mitochondrial DNA data related to the analyses conducted during this study are available on GenBank under accession numbers, as indicated in Table 1.

## Appendix A

**Table A1:** A table showing the sample sizes and diversity indices for the three gecko species analysed during this study, for each respective gene. Data are based on the smallest homologous sequence per species.

	*Hemidactylus turcicus*	*Tarentola mauritanica* Clade III	*Tarentola fascicularis*/*deserti* Clade VIII
*n*	6	21	3
**12S**	393 bp	383 bp	399 bp
number of haplotypes	1	1	1
novel haplotypes	0	0	0
haplotype diversity	0.0000	0.0000	0.0000
nucleotide diversity	0.0000	0.0000	0.0000
**16S**	561 bp	523 bp	526 bp
number of haplotypes	3	1	1
novel haplotypes	3	0	0
haplotype diversity	0.600 ± 0.215	0.0000	0.0000
nucleotide diversity	0.0012 ± 0.0.0012	0.0000	0.0000
**COI**	647 bp	638 bp	658 bp
number of haplotypes	2	3	1
novel haplotypes	2	1	1 ^1^
haplotype diversity	0.333 ± 0.215	0.186 ± 0.110	0.0000
nucleotide diversity	0.0005 ± 0.0007	0.0003 ± 0.0004	0.0000
BOLD BIN	BOLD:ABY0780	BOLD:AAK0390	n/a
**Concatenated data**	1601 bp	1544 bp	1583 bp
number of haplotypes	4	3	1
haplotype diversity	0.800 ± 0.172	0.186 ± 0.110	0.0000
nucleotide diversity	0.0006 ± 0.0006	0.0001 ± 0.0002	0.0000

^1^ Currently there are no publicly available data on COI sequence of this clade.

**Table A2:** The 16S sequence representing different specimens of the *Tarentola mauritanica* complex used for phylogenetic analyses.

Location	GenBank Accession Numbers	References
**Islands in Sicilian Channel**		
**Lampedusa, Italy**	PV715694, PV715695	[18]
**Pantelleria, Italy**	PP338261 (representing 4 individuals)	[17]
**Linosa, Italy**	TMLi1, TMLi2	[21] ^1^
**Other locations** ^2^		
**Morocco**	HM014561, HM014547, HM014548, HM014553, JQ300994, JQ300983, JQ300975, JQ300858, JQ300815, JQ300826, JQ300771, JQ300957, JQ300940, JQ300932, JQ300819, JQ300821, JQ300862, JQ300914, JQ300859, JQ300808, JQ300834, JQ300860, JQ300789, JQ300871, JQ300854, JQ300928	[12,56]
**Algeria**	JQ300783, JQ300875, JQ300946, JQ300788, JQ300901	[12]
**Tunisia**	HM014537, JQ300950	[12,56]
**Spain**	HM014568, HM014562, JQ300976, JQ300822, JQ300816, JQ300836, JQ300908, JQ300907, JQ300988, JQ300812, JQ300980, JQ300962, JQ300931, JQ300987	[12,56]
**France**	JQ300981, JQ300844, JQ300951, JQ300965	[12]
**Italy**	JQ300831, JQ300787, JQ300796, JQ300923, JQ300785, JQ300893, JQ300806, JQ300935, JQ300986, JQ300885, JQ300774	[12]
**Croatia**	JQ300777, JQ300992, JQ300877, JQ300926, JQ300888, JQ300884, JQ300927, JQ300999	[12]
**Canary Islands** ^3^ *(T. angustimentalis)*	JQ300803, JQ300820, JQ300852, JQ300886, JQ300918	[12]
***Tarentola neglecta***	JQ300874 (outgroup)	[12]

^1^ Data obtained from supplementary data of the respective publication [21]. ^2^ Other locations focused on Rato et al. [12,56] to assign clades. ^3^ Data from the Canary Islands was included, given that *Tarentola angustimentalis* occurs within the *T. mauritanica* complex [10,12].

**Table A3:** The 16S sequence representing different specimens of the *Tarentola fascicularis*/*deserti* complex used for phylogenetic analyses.

Location	GenBank Accession Numbers	References
**Islands in Sicilian Channel**		
**Lampedusa, Italy**	FJ609249, FJ609253, FJ609254, FJ609255, HM014555, JQ300776, JQ300811, JQ300823, JQ300840, JQ300841, JQ300855, JQ300856, JQ300870, JQ300872, JQ300882, JQ300910, JQ300995, TMLa1, OR940491, PV715685, PV715686, PV715687, PV715688, PV715689, PV715690, PV715691, PV715692, PV715693	[8,12,18,20,21,56] **^1^**
**Conigli islet, Italy**	FJ609248, FJ609250, FJ609251, FJ609252, HM014545, HM014546, JQ300814, JQ300827, JQ300847, JQ300879, JQ300905	[8,12,56]
**Pantelleria, Italy**	PP338262	[17]
**Chergui Island, Tunisia**	JQ300770, JQ300800	[12]
**Other locations ^2^**		
**Morocco**	JQ300857, JQ300866, JQ300868, JQ300880, JQ300917, JQ300920, JQ300921, JQ300924, JQ300925, JQ300930, JQ300934, JQ300971, JQ300977, JQ300979, JQ300996	[12]
**Algeria**	JQ300772, JQ300775, JQ300805, JQ300813, JQ300835, JQ300863, JQ300864, JQ300896, JQ300904, JQ300909, JQ300911, JQ300938, JQ300949, JQ300956, JQ300964, JQ300967, JQ300969, JQ300990	[12]
**Tunisia**	JQ300832, JQ300839, JQ300892, JQ300916, JQ300933, JQ300953, JQ300958, JQ300984, JQ300985	[12]
**Libya**	JQ300780, JQ300828, JQ300829, JQ300838, JQ300876, JQ300889, JQ300890, JQ300891, JQ300941, JQ300968, JQ300982	[12]
**Egypt**	JQ300791, JQ300961	[12]
***Tarentola neglecta***	JQ300874 (outgroup)	[12]

^1^ Data obtained from supplementary data of the respective publication [21]. ^2^ Other locations focused on Rato et al. [12] to assign clades.
